# Cardiac arrest following blunt trauma-induced tension viscerothorax mimicking tension pneumothorax: A rare case report

**DOI:** 10.1097/MD.0000000000040750

**Published:** 2025-01-03

**Authors:** Zhuo Yuan, Changsheng Liao, Songtao Zhang, Aiwen Wang, Congcong Zhou, Wenbin Yi, Zehao Han, Shaoxiong Xue, Xuefeng Shen

**Affiliations:** a Department of Emergency, Changzhi People’s Hospital, Affiliated with Changzhi Medical College, Changzhi, Shangxi, China; b Department of Orthopedics, Changzhi Peace Hospital, Affiliated with Changzhi Medical College, Changzhi, Shangxi, China; c Department of Henan University of Science and Technology, Luoyang, Henan, China.

**Keywords:** bedside point-of-care ultrasound, blunt trauma, cardiac arrest, case report, tension pneumothorax, tension viscerothorax

## Abstract

**Rationale:**

Tension viscerothorax is a severe condition characterized by significant increases in thoracic pressure due to the herniation of abdominal organs into the thoracic cavity. It is commonly observed in children with congenital diaphragmatic hernias or as a postoperative complication, while tension viscerothorax resulting from blunt trauma is rare.

**Patient concerns:**

A 48-year-old male was urgently admitted to the emergency department with dyspnea following a fall from a height of 15 m.

**Diagnoses:**

The patient, presenting in shock and based on clinical signs, was initially diagnosed with a tension pneumothorax (TPT). Bedside point-of-care ultrasound (POCUS) revealed substantial parenchymal echo abnormalities in the left thoracic cavity and cardiac displacement to the right, suggesting a left-sided tension viscerothorax. Thoracic and abdominal computed tomography confirmed the diagnosis of a rare left-sided tension viscerothorax.

**Interventions:**

Due to the delayed diagnosis, the patient experienced a cardiac arrest. Following cardiopulmonary resuscitation and advanced life support, the patient regained spontaneous circulation and underwent an emergency laparotomy to reduce abdominal organs and repair a diaphragmatic hernia. Postoperatively, the patient received comprehensive medical care.

**Outcomes:**

The patient recovered well postsurgery and was discharged after an 18-day hospital stay. Follow-up over 2 years revealed no significant complications.

**Lessons:**

Blunt trauma-induced tension viscerothorax is rare and can easily be confused with TPT, leading to misdiagnosis. Early use of bedside POCUS is recommended for suspected cases to expedite identification and management, thereby improving survival rates.

## 1. Introduction

Tension viscerothorax is a severe condition where abdominal organs herniate into the thoracic cavity, leading to a significant increase in intrathoracic pressure. This can compress the heart and lungs, impairing normal respiratory and circulatory function. It is a rare complication of diaphragmatic rupture.^[1]^ The clinical manifestations of tension viscerothorax and tension pneumothorax (TPT) are similar, necessitating differential diagnosis. We report a case of tension viscerothorax with an initial delayed diagnosis, where the patient experienced hemodynamic deterioration and cardiac arrest. Following successful resuscitation, the patient underwent surgical treatment and ultimately recovered and was discharged.

## 2. Case presentation

A 48-year-old man was admitted to the emergency department 2 hours after falling from a height of 15 m, On admission, physical examination revealed a body temperature of 36.2°C, heart rate of 150 beats per minute, weak radial pulse, respiratory rate of 35 breaths per minute, blood pressure of 70/35 mm Hg, oxygen saturation of 89%, restlessness, shortness of breath, multiple skin abrasions across the body, and significant neck pain. The left chest wall was swollen, with subcutaneous emphysema and absent breath sounds on the left lung. The abdominal muscles were tense with abdominal tenderness and rebound pain. The left hip was tender with deformity and shortening of the left lower limb. Laboratory tests showed: arterial blood gases: pH 7.21, PO_2_ 53 mm Hg, PCO_2_ 28 mm Hg, lactate 3.6 mol/L; complete blood count: white blood cells 22.77 × 10^9^/L, hemoglobin 114 g/L, hematocrit 33.6%, platelet count 233 × 10^9^/L; coagulation profile: prothrombin time 17.8 seconds, international normalized ratio 1.57, activated partial thromboplastin time 42.2 seconds; liver and kidney functions were normal. The timeline of interventions summarizing key events from the initial presentation to the patient’s recovery is shown (Table 1).

**Table 1:** The timeline of interventions for the patient.

Time	Event
2 h before admission	The patient fell from a height of 15 m and presented with dyspnea.
After admission	The patient exhibited signs of shock, including hypotension, agitation, and tachypnea. Physical examination revealed multiple skin abrasions, swelling in the neck, and subcutaneous emphysema on the left side. Absent breath sounds were noted in the left lung. Additionally, the abdominal muscles were tense, with tenderness and rebound tenderness present.
5 min later	Considering the possibility of tension pneumothorax, an urgent left-sided chest drain was performed, which yielded only a small amount of dark red fluid and gas. Concurrently, vasoactive drugs were administered, and mechanical ventilation support was initiated.
8 min later	Bedside ultrasound suggested tension viscerothorax.
10 min later	CT scan confirmed the diagnosis of tension viscerothorax.
15 min later	The patient experienced cardiac arrest and was immediately resuscitated.
20 min later	Cardiac resuscitation was successful.
1 h later	Urgent surgery was performed to relieve cardiac compression and repair the diaphragmatic injury.
1 d later	Follow-up CT of the chest and abdomen showed good condition.
18 d later	The patient was discharged, demonstrating good recovery.
6 mo later	Follow-up chest CT indicated no symptoms, and the patient resumed pre-injury physical activity levels.
2 yr later	Follow-up chest X-ray showed no symptoms, and the patient’s health status was good.

CT = computed tomography.

**Figure 1.**
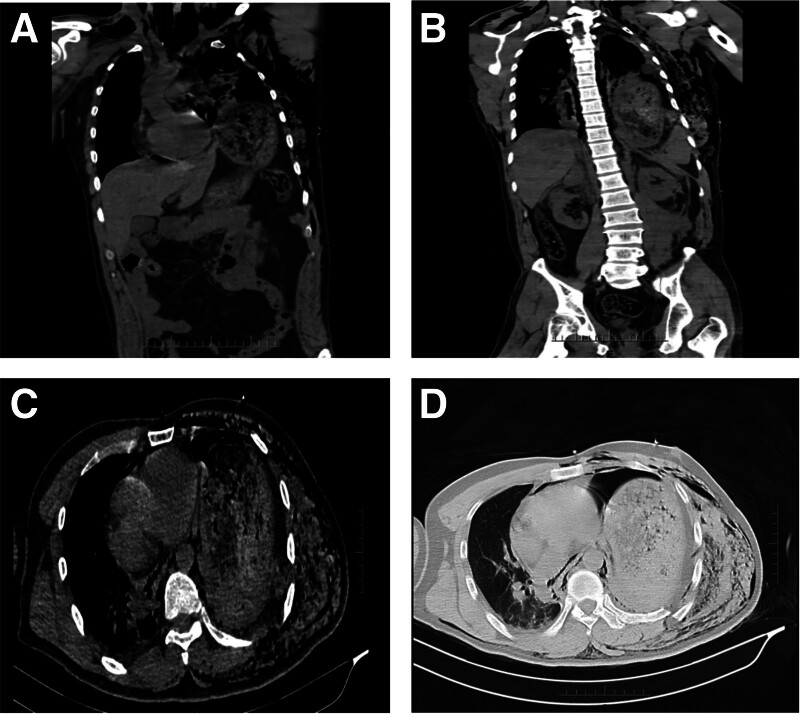
Patient’s chest and abdominal CT. (A) Stomach herniation into the left thoracic cavity. (B) Stomach and spleen above the diaphragm. (C) Left-sided tension viscerothorax with mediastinal and cardiac rightward shift. (D) Left-sided tension viscerothorax, mediastinal and cardiac rightward shift, left rib fractures, and significant left lung compression. CT = computed tomography.

The patient exhibited symptoms of shock, and given the clinical signs, traumatic acute left-sided tension pneumothorax was highly suspected. Immediate closed chest drainage was performed on the left side, which only drained a small amount of dark red fluid and air, suggesting other severe injuries. The patient received fluid resuscitation, norepinephrine infusion (3–6 mg/hour) to maintain blood pressure, and noninvasive ventilator support. His heart rate stabilized between 140 and 160 beats per minute, blood pressure between 90-100/50-67 mm Hg, and oxygen saturation between 95% and 98%. Bedside point-of-care ultrasound (POCUS) revealed substantial echogenic abnormalities within the left thoracic cavity and rightward displacement of the heart, suggesting left-sided tension viscerothorax. To confirm the diagnosis, an urgent chest and abdominal computed tomography (CT) scan was performed (Fig. 1).

**Figure 2.**
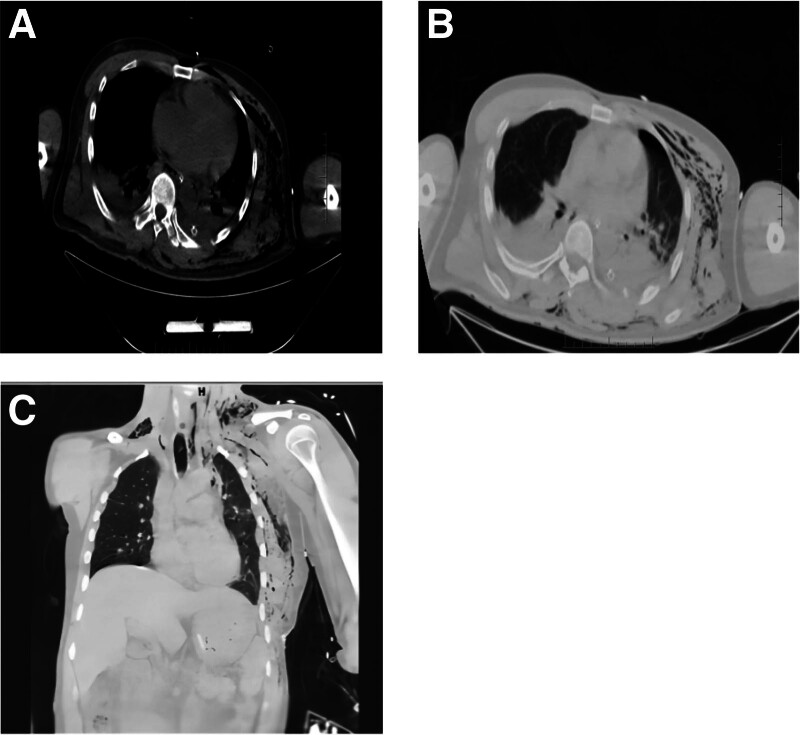
Postoperative chest and abdominal CT. (A, B) Small bilateral pleural effusion, left lower lung consolidation. (C) Postoperative stomach and spleen repositioned in the abdominal cavity, mediastinal and cardiac repositioning. CT = computed tomography.

Upon returning to the resuscitation room after the CT scan, the patient suddenly lost consciousness, was unresponsive when called, and his carotid pulse disappeared, indicating cardiac arrest. Immediate cardiopulmonary resuscitation and advanced life support were initiated, and the patient regained spontaneous circulation after 5 minutes. He was then urgently taken to the operating room. Emergency surgery repaired the lung laceration, diaphragmatic rupture, reduced left rib fractures, and repositioned the stomach, omentum, spleen, and part of the left liver lobe into the abdominal cavity, and a left-sided chest drain was placed. Postoperative chest CT scans were performed (Fig. 2). The patient was discharged on the 18th day of hospitalization, showing satisfactory recovery.

**Figure 3.**
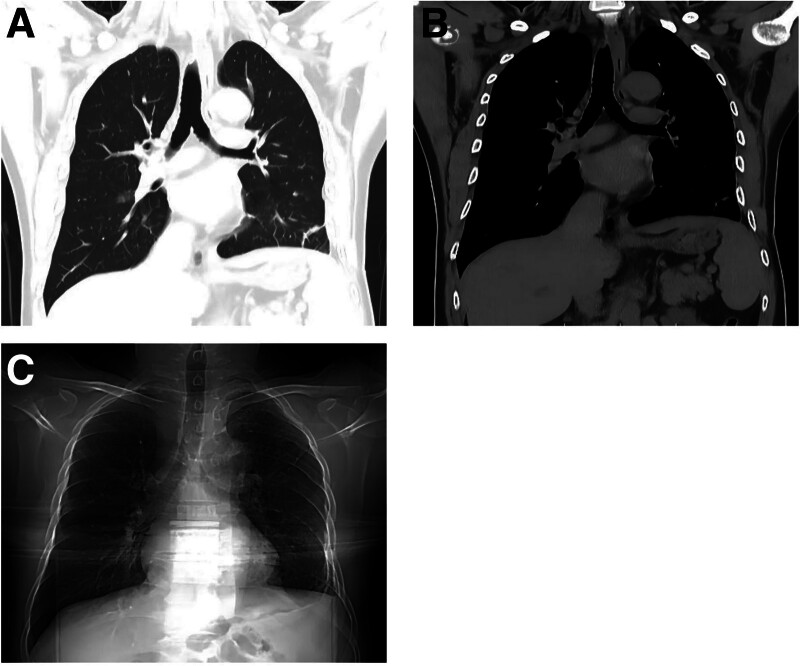
Follow-up imaging results. (A, B) Six months postoperatively, chest CT shows bilateral lungs essentially normal, with the liver, spleen, and stomach in their proper anatomical positions. (C) Two-year postoperative follow-up chest X-ray reveals no significant abnormalities. CT = computed tomography.

The patient was closely monitored for 2 years postdischarge. The first follow-up occurred 6 months after discharge, during which a chest CT scan was performed (Fig. 3). At this time, the patient reported no symptoms and was able to engage in physical activities at pre-injury levels. The second follow-up took place 2 years postdischarge. This consistent monitoring confirms that the patient has had a favorable long-term outcome, with no complications related to the initial injury. The patient remained asymptomatic, and a chest X-ray conducted at this follow-up also showed normal findings (Fig. 3).

## 3. Discussion

In this case, the patient was admitted after falling from a height, presenting with shock symptoms, initially resembling traumatic acute left-sided TPT. Considering the urgency of TPT, left-sided closed thoracic drainage was immediately performed. However, only a small amount of dark red fluid and gas was drained, indicating other severe injuries, such as rib fractures puncturing the lungs or heart vessels, direct injury to the heart or lungs, or diaphragmatic rupture. Given the high risk of multiple organ injuries from a high fall, a comprehensive evaluation of potential injuries, including intracranial hemorrhage, liver and spleen rupture, and multiple fractures, is crucial. Given the patient’s hemodynamic instability and high risk of cardiac arrest, excessive movement for a full-body CT scan posed high risks. Therefore, POCUS was chosen, revealing a solid abnormal echo in the left thoracic cavity with rightward cardiac displacement, further supporting the tension viscerothorax diagnosis. While providing emergency treatment, fluid resuscitation, blood pressure support, and noninvasive ventilatory support stabilized the patient’s vital signs. Subsequently, an emergency thoracoabdominal CT scan was performed to confirm the diagnosis, ensuring appropriate treatment for all potential internal injuries, maximizing the patient’s survival chances and treatment effectiveness.

Acute diaphragmatic rupture occurs in 1% to 7% of blunt trauma patients,^[2]^ but tension viscerothorax formation is rare. The clinical presentation of tension viscerothorax closely resembles that of TPT, often leading to misdiagnosis.^[1]^ During the initial assessment, clinicians should carefully evaluate the patient’s history of blunt trauma, as tension viscerothorax is commonly associated with severe abdominal injuries or diaphragmatic rupture, while tension pneumothorax often results from rib fractures or chest trauma. A thorough physical examination is essential, as tension viscerothorax can present with abdominal findings such as rigidity and tenderness, alongside potential respiratory signs like decreased breath sounds and ipsilateral chest wall expansion, which may also occur in tension pneumothorax. Despite both conditions causing obstructive shock, treatment differs significantly. The first-line treatment for TPT is immediate closed thoracic drainage.^[3,4]^ However, for tension viscerothorax, blind thoracic puncture may result in fatal gastric or intestinal perforation.^[5]^ Hence, early accurate diagnosis is crucial.

Diaphragmatic rupture on imaging has a sensitivity of up to 94%.^[6]^ If hollow organs herniate, bowel loops in the thoracic cavity may be seen on X-rays.^[7]^ Ahn et al^[1]^ reported a 10-year-old boy diagnosed with tension viscerothorax via portable chest X-ray. Studies have shown promising results in using artificial intelligence models to differentiate between pneumothorax and tension pneumothorax through X-ray imaging. Developing AI models to distinguish tension viscerothorax from tension pneumothorax may be a future direction.^[8]^ POCUS has proven valuable in diagnosing diaphragmatic hernias, detecting solid or cystic echoes in the thoracic cavity.^[9]^ Additionally, Olusanya and Lashin^[10]^ diagnosed a case of tension pneumothorax using ultrasound, further validating the value of ultrasound in such assessments. In this case, POCUS revealed cardiac rightward shift and solid abnormal echoes in the left thoracic cavity, suggesting tension viscerothorax. Excessive handling of unstable patients with severe multiple injuries may increase mortality, so POCUS is recommended for initial diagnosis. CT scanning can rapidly provide detailed anatomical information, revealing the presence of herniated abdominal organs, diaphragmatic rupture, and any associated intrathoracic fluid or air. This swift identification can significantly reduce the time required for both diagnosis and intervention. Therefore, for stable patients, CT scanning is the gold standard. However, while CT is considered the gold standard, other imaging modalities, such as X-rays and ultrasound, also have their applications in specific contexts. For instance, X-rays can serve as an initial assessment tool, although their sensitivity in detecting subtle injuries is comparatively low. Ultrasound offers the advantage of being quick and suitable for bedside use; however, its effectiveness can be compromised by the presence of intra-abdominal gas or skin trauma, which may hinder accurate identification of underlying pathology. Maya Horst et al^[11]^ reported a female infant with respiratory distress and unstable vitals misdiagnosed with TPT via X-ray. After chest drainage improved symptoms, CT confirmed tension viscerothorax. The infant underwent diaphragmatic repair surgery and recovered. CT scanning reduces misdiagnosis and reveals hidden diaphragmatic lesions in stable patients. Although magnetic resonance imaging offers high tissue contrast and resolution, its long acquisition time and need for patient cooperation limit its use in critically ill patients.^[9]^

In Shin Ahn et al’s case, tension viscerothorax patient initially failed nasogastric tube insertion, and unstable vitals were not promptly corrected, leading to cardiac arrest. Fortunately, successful resuscitation and second successful nasogastric tube insertion released about 1600 mL of gastric contents, stabilizing blood pressure, and the patient was discharged postsurgery.^[1]^ If nasogastric tube insertion had failed again, survival was unlikely.^[1]^ In the case reported by Biloslavo et al,^[12]^ the patient was diagnosed with tension viscerothorax through X-ray and CT imaging. During the waiting period for surgical intervention, the patient experienced cardiac arrest. Following successful cardiopulmonary resuscitation, surgical intervention was performed to relieve cardiac compression, leading to the patient’s eventual recovery and discharge. Therefore, once tension viscerothorax is diagnosed, it is crucial to relieve the compression and stabilize vital signs, followed by surgical treatment to restore the thoracoabdominal anatomy.

## 4. Conclusion

In summary, tension viscerothorax resulting from blunt trauma is extremely rare, and its clinical presentation closely resembles that of tension pneumothorax, often lacking specific distinguishing features that can lead to misdiagnosis. Therefore, clinicians should rigorously assess the patient’s history, symptoms, and physical findings in cases of blunt trauma, remaining vigilant for the possibility of tension viscerothorax. For patients with unstable vital signs and challenging differential diagnoses, early utilization of bedside ultrasound is recommended for timely diagnosis. Upon confirming tension viscerothorax, prompt surgical intervention is essential to enhance patient survival and reduce the risk of complications.

## Acknowledgments

We thank Dr Bin Qiao for editing this manuscript.

## Author contributions

**Conceptualization:** Zhuo Yuan, Aiwen Wang, Xuefeng Shen.

**Formal analysis:** Zhuo Yuan, Aiwen Wang.

**Investigation:** Changsheng Liao, Congcong Zhou.

**Methodology:** Zhuo Yuan, Songtao Zhang, Aiwen Wang.

**Resources:** Aiwen Wang.

**Validation:** Zhuo Yuan, Xuefeng Shen.

**Visualization:** Wenbin Yi, Zehao Han, Shaoxiong Xue.

**Writing – original draft:** Zhuo Yuan, Xuefeng Shen.
